# Controlled Formation of Carboxymethyllysine in Bone Matrix through Designed Glycation Reaction

**DOI:** 10.1002/jbm4.10548

**Published:** 2021-10-19

**Authors:** Grażyna E. Sroga, Deepak Vashishth

**Affiliations:** ^1^ Department of Biomedical Engineering Rensselaer Polytechnic Institute, Center for Biotechnology and Interdisciplinary Studies Troy NY USA

**Keywords:** BONE MATRIX, CARBOXYMETHYLLYSINE, DESIGNED IN VITRO GLYCATION, HUMAN, OXIDATIVE/CARBONYL STRESS

## Abstract

It has been a challenge to establish a link between specific advanced glycation end products (AGEs) as causal agents of different pathologies and age‐related diseases, primarily because of the lack of suitable in vitro experimental strategies facilitating increased formation of a specific AGE, here carboxymethyllysine (CML), over other AGEs under controlled conditions. CML is of considerable importance to various oxidative stress–related diseases, because in vivo formation of this AGE is connected with cellular oxidative/carbonyl metabolism. The mechanistic implications of CML accumulation in bone remain to be elucidated. To facilitate such studies, we developed a new in vitro strategy that allows preferential generation of CML in bone matrix over other AGEs. Using bone samples from human donors of different age (young, middle‐age, and elderly), we show successful in vitro generation of the desired levels of CML and show that they mimic those observed in vivo in several bone disorders. Formation of such physiologically relevant CML levels was achieved by selecting two oxidative/carbonyl stress compounds naturally produced in the human body, glyoxal and glyoxylic acid. Kinetic studies using the two compounds revealed differences not only between their reaction rates but also in the progression and enhanced formation of CML over other AGEs (measured by their collective fluorescence as fluorescent AGEs [fAGEs]) Consequently, through the regulation of reaction time, the levels of CML and fAGEs could be controlled and separated. Given that the developed approach does not fully eliminate the formation of other uncharacterized glycation products, this could be considered as the study limitation. We expect that the concepts of our experimental approach can be used to develop diverse strategies facilitating production of the desired levels of selected AGEs in bone and other tissues, and thus, opens new avenues for investigating the role and mechanistic aspects of specific AGEs, here CML, in bone. © 2021 The Authors. *JBMR Plus* published by Wiley Periodicals LLC on behalf of American Society for Bone and Mineral Research.

## Introduction

Except for certain anaerobic and oxygen‐tolerant organisms (also termed “air‐tolerant”; they are described as organisms killed by atmospheric concentration of oxygen of 21%, but depending on a species are capable of surviving from less than 0.5% to as much as 8% oxygen) all microbes, plants, and animals require oxygen for efficient production of energy. They use oxygen‐dependent electron‐transport pathways such as those in the mitochondria of mammalian cells, which produce over 80% of the key energy‐storing molecule adenosine triphosphate. The need for oxygen obscures the fact that O_2_ is a toxic mutagenic gas, and aerobes survive because they have antioxidant defenses to protect against it. The damaging effect of O_2_ is usually caused by the oxidation of essential cellular components (eg, proteins, nucleic acids, lipids) and a decrease in the pool of reducing equivalents needed for biological reactions within a cell. However, these reactions often simultaneously reduce O_2_ to free oxygen radicals and other oxygen‐derived toxic compounds collectively known as reactive oxygen species (ROS). These are, for example, superoxide, hydrogen peroxide, and hydroxyl radical ions, and typically they lead to oxidative stress. Of importance to this work is that oxidative stress is involved in the formation of a large subgroup of advanced glycation end products (AGEs) known as glycoxidation end products (AGOEs).

Formation of AGEs and AGOEs is of great interest to health and medicine because they are formed at a slow but constant rate in a healthy human body beginning at early embryonic development and continue to accumulate with time, altering key processes and functions. The term AGEs/AGOEs is applied to a broad range of the Maillard reaction products including N^ε^‐(1‐carboxymethyl)‐L‐lysine (also known as N(6)‐carboxymethyllysine or carboxymethyllysine [CML]),^(^ ^1^, ^2^ ^)^ pentosidine (a mature fluorescent crosslink accumulating with age that was the first chemically characterized AGE^(^ ^3^ ^)^), glucosepane,^(^ ^4^ ^)^ vesperlysines,^(^ ^5^ ^)^ and a number of other compounds.^(^ ^6^, ^7^ ^)^ Concentrations of AGEs vary depending on the tissue and AGE type. Glycation products are formed from a wide range of carbohydrates (eg, hexoses, pentoses, tetroses),^(^ ^6^, ^7^, ^8^ ^)^ different carbohydrate precursors^(^ ^6^, ^9^ ^)^ and glucose degradation products such as 3‐deoxyglucosone, methylglyoxal, glyoxal, formaldehyde, or acetaldehyde. Aldehydes (eg, glucose, formaldehyde) and ketones (eg, fructose, acetoacetate) are important biological reagents that covalently modify proteins. Notably, glucose degradation products, which are generated in a body by a variety of enzymatic and nonenzymatic processes, are more potent precursors for AGE/AGOEs formation than glucose itself.^(^ ^8^, ^10^ ^)^ In addition, short chain aldehydes (eg, formaldehyde) and ketones (eg, acetoacetate) are formed in a body independently from glucose oxidation through different metabolic processes such as lipid oxidation, formation of ROS and their slow removal that could be attributed to the decline of efficiency of detoxifying processes ^(^ ^11^, ^12^ ^)^ (eg, caused by aging and diseases). Except for ~45 structurally defined AGEs generated in vivo, the chemical identity of many other AGEs is currently unknown. One of the reasons for such limited information is that AGEs form a large group of complex and very heterogeneous compounds (eg, a large subgroup of AGEs measured by their collective fluorescence and known as fluorescent AGEs [fAGEs] is regularly used to characterize glycation products in different tissues) Thus, despite intensive studies, the mechanisms that generate diverse glycation products remain elusive. The mechanistic aspects of AGE effects on various tissues (eg, on their homeostasis and function) have been hindered by the lack of suitable in vitro experimental tools elevating the formation of a specific AGE of interest over other AGEs under controlled in vitro conditions. This research gap has been addressed by our study, which aimed to enhance CML formation over other AGEs.

Considering skeletal tissue, the studies on the role and mechanistic aspects of specific AGEs in, for example, bone cell responses^(^ ^13^, ^14^, ^15^ ^)^ or biomechanical properties of bone,^(^ ^16^ ^)^ rely considerably on the in vitro methods. These studies have also been hindered by the lack of suitable in vitro experimental approaches enhancing formation of specific AGEs. Hence, we aimed to develop a strategy elevating formation of CML over other AGEs in the extracellular matrix (ECM) of bone. We sought to generate the CML levels of clinical relevance, for example, the levels that would correspond to those observed in vivo in such conditions as aging,^(^ ^2^, ^17^, ^18^ ^)^ diabetes,^(^ ^2^, ^19^ ^)^ and renal failure,^(^ ^19^ ^)^ to investigate bone properties in different skeletal‐related disorders including—but not limited to—osteoporosis, type 1 and type 2 diabetes mellitus (T1DM and T2DM), or osteosarcoma.

**Fig 1:**
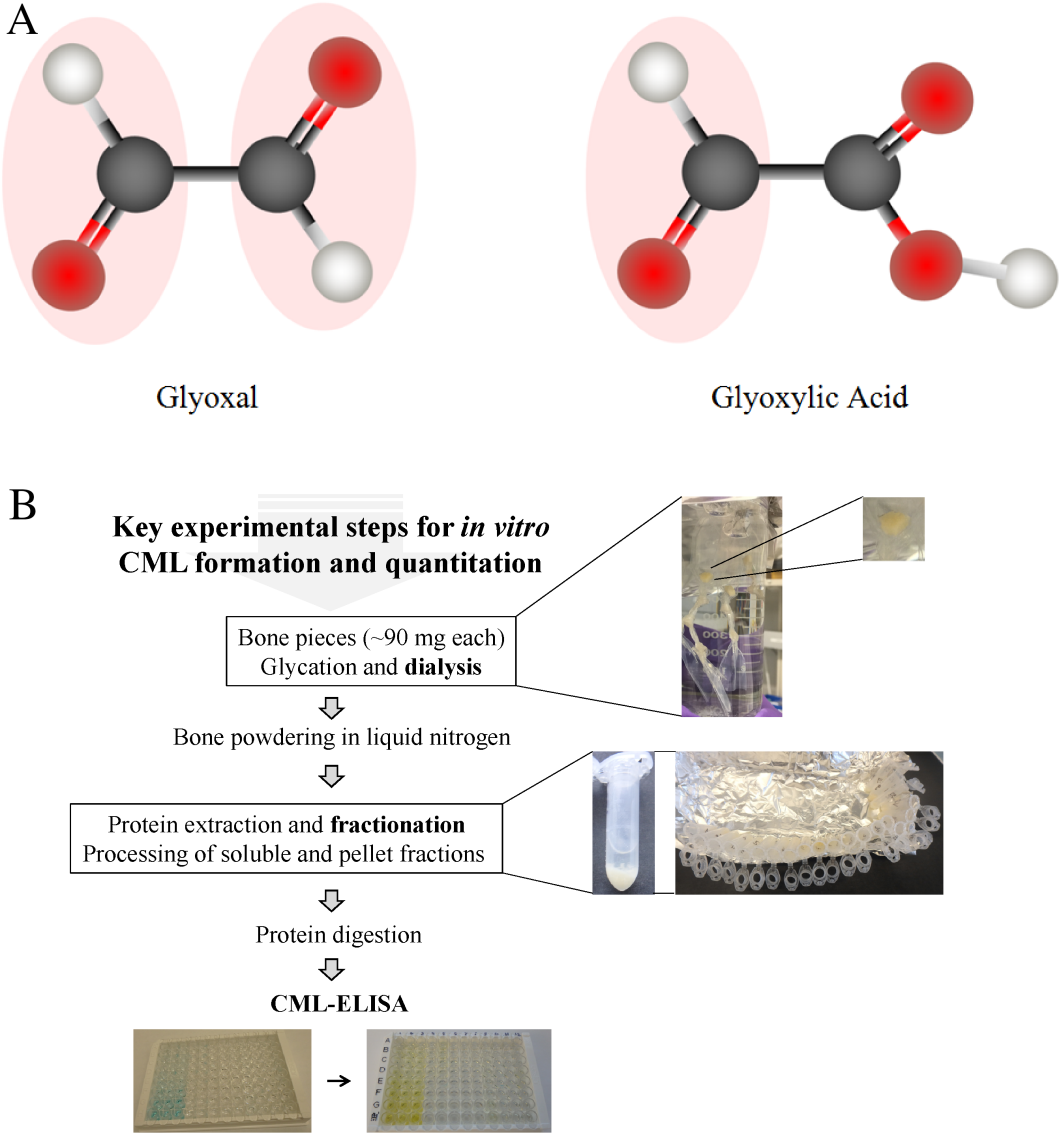
(*A*) Structure of glyoxal (contains two aldehyde groups) and glyoxylic acid (contains one aldehyde group) showing the reactive aldehyde groups (–CHO; marked in pink). Ball color code: carbon = black, oxygen = red, hydrogen = gray. (*B*) Schematic showing the work flow for the key experimental steps of CML formation and quantitation. Description of each step is detailed in the Materials and Methods section.

CML is a non‐fluorescent AGE that has recently been shown to accumulate in human bone^(^ ^20^ ^)^ and correlate with bone fracture properties^(^ ^16^ ^)^ and fracture incidences.^(^ ^21^ ^)^ This AGE can be formed in vivo through a number of different glycation and glycoxidation pathways. Depending on the disease type, formation of AGEs can be shifted toward distinct glycoxidation pathways. Typical condensation of glucose with the ε‐amino group of lysine results in the formation of fructoselysine, an Amadori rearrangement product that is subsequently oxidized to form CML. Of importance to this study is one of the alternative in vivo oxidative routes that leads to the formation of CML through a reaction of glyoxal with lysine residues.^(^ ^22^, ^23^, ^24^, ^25^, ^26^, ^27^ ^)^ Glyoxal as well as other products of oxidative/carbonyl stress (eg, glycolaldehyde, methylglyoxal) are produced in vivo during lipid peroxidation and sugar degradation.^(^ ^23^, ^25^, ^26^ ^)^ Furthermore, ~40% to 50% of glyoxal originates from a pre‐Amadori reaction step largely independent of glucose autoxidation. This step is also known as Namiki pathway of the Maillard reaction.^(^ ^27^, ^28^, ^29^ ^)^ In addition to the in vivo formation of glyoxal, the general human population is typically exposed to glyoxal, glyoxylic acid, and glycolic acid through drinking water, intake of food, and the use of cosmetics. Glyoxal, glyoxylic acid, and glycolic acid^(^ ^30^ ^)^ are also present in tobacco smoke, residential log fire smoke, and vehicle exhausts. Concentration of glyoxal in human blood is in the range of 0.1 to 1.0 μmol/L (0.006–0.06 mg/L) and is markedly higher in T2DM and renal failure.^(^ ^31^ ^)^ Due to the connection of the in vivo CML formation with oxidative/carbonyl stress, this AGE is of considerable importance to T2DM, renal failure, and other oxidative stress–related diseases. These considerations and the aforementioned natural route of CML formation (ie, reaction of glyoxal and glyoxylic acid with the lysine residues through the Namiki pathway) motivated the development of our in vitro strategy to specifically enhance the formation of CML over other AGEs in ECM of bone using glyoxal and glyoxylic acid (Fig. 1*A*).

To this end, we evaluated several experimental approaches and developed a new in vitro strategy that enhances the formation of CML in bone's ECM using mineralized bone samples from different human donors. The developed strategy facilitates formation of the desired levels of CML in bone. Thus, it can be used, for example, to mimic or increase CML levels that are observed in vivo in various bone and bone‐related disorders, including those that are determined in vivo in human aging and/or diabetic bone. We expect that our strategy could open new avenues for diverse studies on the role of this specific AGE (ie, CML) in bone and other tissues, as well as could serve as a prerequisite for the development of diverse in vitro strategies enhancing controlled formation of other specific AGEs. As such, it could also open new avenues for investigating the role and mechanistic aspects of an array of specific AGEs.

## Materials and Methods

### Bone samples

Tibiae (posterior area) from three age groups of donors (young, 25‐year‐old Caucasian male [CM] [25 CM]; middle age, 61‐year‐old CM [61 CM]; and elderly, 89‐year‐old Caucasian female [CF] [89 CF]) served as the source of cortical bone tissues. The reason for selection of samples from donors of such a pronounced age difference (~30‐year sequential gap) was to determine how the quality of bone matrix and mineral, known to change with age,^(^ ^8^, ^32^ ^)^ would influence the development of the in vitro reaction conditions and the overall glycation process. When compared to other tissues, biochemical analyses of bone face a number of additional challenges that are attributed to its unusual composition. Bone contains a large amount of a mineral (60%–80%), the content of which varies with bone type, anatomical bone/skeleton site, disease, species, and drug treatment^(^ ^33^ ^)^; a small amount of total organic material (10%–20%) that contains a uniquely large proportion of collagen (~90%)^(^ ^34^ ^)^; and water (10%).^(^ ^35^ ^)^ All tibia specimens were obtained from the centralized National Disease Research Interchange (NDRI) biobank and were known to be free of osteoarthritis, diabetes, and other metabolic bone diseases as well as human immunodeficiency virus (HIV) and hepatitis B virus (HBV).

Three separate bone samples from each donor of ~90 to 100 mg in weight were cut into a shape of a cube (Fig. 1*B*; a photograph showing an example of bone cubes that are visible inside a dialysis tube; Repligen, Rancho Dominguez, CA, USA) using a slow‐speed diamond blade (Buehler, Lake Bluff, IL, USA). The pieces were repeatedly washed in cold distilled water until the washings were free of blood, and then, defatted three times for 15 minutes using 2 mL of isopropyl ether. Depending on the experimental plan, fresh or freeze‐dried bone specimens were stored at −80°C until further use. To freeze‐dry samples, the FreeZone 6 liter benchtop freeze‐dry system with polytetrafluoroethylene (PTFE)‐coated collector (Labconco, Kansas City, MO, USA) was used. Just before placing glass containers with the samples into the machine outlets, the vials were covered with Parafilm®M (Millipore Sigma, St. Louis, MO, USA) that was next punctured in a few places, and cooled down in liquid nitrogen. The process of freeze‐drying was conducted overnight.

### In vitro enhancement of Nε‐(1‐carboxymethyl)‐L‐lysine formation in bone matrix

Bone samples (in triplicate) designated for glycoxidation were placed into vials with cyanoborohydride coupling buffer (0.20M disodium phosphate, pH 8.0–8.5, 0.20M NaCl, 3.0 g/L sodium cyanoborohydride; all buffer components were purchased from Sigma‐Aldrich, St. Louis, MO, USA) supplemented with 0.15M glyoxal or 0.15M glyoxylic acid (Fig. 1*A*) and 0.5mM CaCl_2_, and incubated at 37°C for 24, 48, or 72 hours. The matching control bone samples were incubated at 37°C for 24, 48, or 72 hours in the 0.20M disodium phosphate pH 8.0 to 8.5, 0.20M NaCl, and 0.5mM CaCl_2_ buffer. After completion of incubations, all samples underwent dialysis against phosphate buffered saline of pH 7.4. In the next experimental step, the samples were lyophilized overnight and stored at −80°C until their analysis.

### Protein isolation

First, each cleaned and defatted bone sample was powdered in liquid nitrogen using a mortar and a pestle.^(^ ^36^ ^)^ Extracellular bone matrix proteins were isolated using a modified procedure described by Sroga and colleagues.^(^ ^36^ ^)^ Briefly, the extraction buffer (0.05M EDTA, 4M guanidine chloride, 30mM Tris‐HCl, 15% glycerol, and 10 μL/mL of Halt Protease Inhibitor from Pierce Biotechnology, Inc./Thermo‐Fisher Scientific [Waltham, MA, USA], pH 7.4) was added directly into the tubes containing powdered bone samples. Simultaneous protein isolation and demineralization was performed for 72 hours at 0°C to 2°C using microdialysis membrane (Spectra Por® 3 Dialysis Membrane; Spectrum Laboratories, Inc., Rancho Dominguez, CA, USA). The resulting protein extracts were fully demineralized as determined by visual inspection using a standard binocular compound microscope B250C (United Scope LLC dba AmScope, Irvine, CA, USA) and manual inspection by aspirating the protein extract through a pipette tip. Subsequently, protein extracts were dialyzed against several changes of the PBS buffer pH 7.4. After dialysis, the samples were centrifuged for 30 minutes at 4°C and 14,000 rpm/20,800*g* (Eppendorf centrifuge model 5417R, rotor F45‐30‐11; Eppendorf North America, Framingham, MA, USA) Both soluble protein fraction (supernatants) and collagen pellets were collected and used for quantitation of CML (Fig. 1*B*; photographs depicting an example of a tube with two protein fractions as well as the tubes with protein pellets after a collection of soluble fractions which are prepared for the next steps of the experiments).

Solubilization of collagen pellets was achieved through a sequential freezing‐and‐thawing procedure (60 minutes at −80°C and 15 minutes at +85°C) to increase protein yield and expedite enzymatic digestion. This was followed by a sequential sonication using Branson sonicator (Branson Ultrasonic Corp., Danbury, CT, USA) (90 minutes sonication and 20 minutes on ice) until ~50 to 60 μg of protein (Table 1) was obtained. This amount was sufficient to perform the CML‐ELISA assay in triplicate.

**Table 1:** Yields of Solubilized Pellet Fractions Containing Collagen and Other Bone Matrix Proteins

Pellet solubilization step	Amount of solubilized proteins (μg)	
	Without vortexing	With vortexing
I: Freezing and thawing		
Cycling		
5× FTC for 1 day (~6.5 hours total)	2.35 ± 0.49	3.25 ± 1.77
10× FTC for 2 days (~13 hours total)	3.25 ± 1.20	4.65 ± 0.64
15× FTC for 3 days (~20 hours total)	5.10 ± 1.56	6.05 ± 1.48
20× FTC for 4 days (~26.5 hours total)	~8.2	~10.1
II: Sonication and cooling		
Cycling		
4× SCC for 1 day (~8 hours total)		24.60 ± 13.72
8× SCC for 2 days (~16 hours total)		45.70 ± 15.70
12× SCC for 3 days (~24 hours total)		68.35 ± 17.32

One freezing and thawing cycle (1× FTC) comprises 60 minutes of incubation at −80°C followed by 15 minutes incubation at +85°C. One sonication and cooling cycle (1× SCC) comprises 90 minutes of sonication followed by 20 minutes incubation on ice. Vortexing of the samples between the cycles for 1 minute refers to the FTC experiments. The ± values depict SD. FTC = freezing and thawing cycle; SCC = sonication and cooling cycle.

### Quantitation of Nε‐(1‐carboxymethyl)‐L‐lysine using CML‐ELISA

Protein concentration in each soluble protein fraction and solubilized collagen sample was measured using a Bradford assay with a bovine serum albumin (BSA) standard per instructions included with the kit (Pierce™ Coomassie Plus™ [Bradford] Protein Assay; Thermo Fisher Scientific, Waltham, MA, USA).

Overnight protein digestion was conducted at 37°C in a buffer (50mM Tris‐HCl, pH 7.4 and 5mM CaCl_2_) containing two proteases, collagenase (collagenase from *Clostridium histolyticum* for general use, Type I; Sigma‐Aldrich, St. Louis, MO, USA) and proteinase K (recombinant, PCR grade; Thermo Fisher Scientific, Waltham, MA, USA).

CML‐ELISA was performed according to the protocol included with the Human Carboxymethyl Lysine (CML) ELISA Kit (MyBioSource, San Diego, CA, USA; catalog number MBS700744). The assay is based on the quantitative sandwich enzyme immunoassay technique where antibody specific for CML is used to pre‐coat the wells of a microtiter (MT) plate. Briefly, standards and samples were pipetted into the wells of a MT plate to allow binding of CML present in the samples to the immobilized antibody. After removing any unbound substances, a biotin‐conjugated antibody specific for CML was added to the wells. Following incubation and a sequence of washings, the avidin‐conjugated horseradish peroxidase (HRP) was next added to the wells. Detection of CML was achieved through an enzymatic reaction performed with HRP in the presence of H_2_O_2_ that oxidizes 3,3′,5,5′‐tetramethylbenzidine (TMB). Oxidized TMB has blue color that changes into yellow at acidic pH when enzymatic reaction is terminated by the addition of the stop solution (0.16M sulfuric acid) (Fig. 1*B*; a photograph showing an example of an MT plate before (blue) and after (yellow) addition of the stop solution). At 450 nm, the color intensity is directly proportional to the CML concentration in the sample. It was measured using a MT plate reader (model Infinite 200; Tecan, Männedorf, Switzerland).

### Measurement of fAGEs

Preparation of the samples and fAGEs measurement was conducted as described by Sroga and colleagues.^(^ ^8^, ^36^, ^37^, ^38^ ^)^ Briefly, direct acid hydrolysis of the glycated bone samples and non‐glycated controls was performed in 6 N HCl (100 μL/mg bone) at 110°C for 20 hours. After completion of hydrolysis, the hydrolysates were centrifuged and the supernatants were divided into two portions. Each portion was transferred into a clean tube and used directly for the assays or stored at −80°C until needed.

The assay to measure fAGEs in bone matrix has two components. The first one is the fluorimetric assay for determination of fAGEs content “in‐bulk.” This assay is based on the measurement of natural fluorescence of AGEs as compared to the fluorescence of the quinine (Q) standards (stock: 10 mg/mL quinine per 0.1 N sulfuric acid) at 360/460 nm excitation/emission using the MT plate reader (model Infinite 200; Tecan).^(^ ^36^, ^37^, ^38^ ^)^ The second assay component is the colorimetric assay for determination of collagen content in bone samples through the measurement of hydroxyproline concentration.

Hydroxyproline was used to prepare the standard curve for the colorimetric assay. All solutions were made fresh directly before their use. The assay was initiated by addition of chloramine‐T solution to hydroxyproline standards (stock: 2 mg/mL L‐hydroxyproline per 0.001 N HCl) and to the hydrolysates of bone samples. These solutions were then incubated at room temperature (RT) for 20 minutes. Subsequently, 3.15M perchloric acid solution was added to the samples and 5 minutes incubation at RT followed. Next, the p‐dimethylaminobenzaldehyde solution was added and the samples were incubated for 20 minutes at 60°C. Finally, all the standards and the samples were cooled down to RT in darkness. The absorbance was measured at 570 nm using the MT plate reader (model Infinite 200; Tecan). Collagen content was calculated based on the determined amount of hydroxyproline.^(^ ^36^, ^37^, ^38^ ^)^ The amount of fAGEs was expressed in the terms of units of fluorescent quinine per unit of collagen (eg, Quinine/Collagen [ng/mg]), which also stands for fAGEs/Total Protein [ng/mg].

### Statistical comparisons

To establish correlations for CML and fAGEs generated using glyoxal and glyoxylic acid, statistical analyses were performed using MATLAB 2021a, Minitab and MS Excel Statistical Analysis ToolPack software (MathWorks, Natick, MA, USA; Minitab, LLC, State College, PA, USA; Microsoft Corp., Redmond, WA, USA). Data analyses and mathematical models derived through different software supplied similar results.

Data are expressed as mean ± standard deviation (SD). To determine if there had been an interaction between the two independent variables (ie, CML or fAGEs formation and donor's age) and the dependent variable (ie, time), two‐factor analysis of variance (ANOVA) with replication α = 0.05 was used, which was followed by a post hoc Tukey's honestly significant difference (HSD) multiple comparison of means (95% confidence interval [CI]) when appropriate. Each main effect and interaction in the ANOVAs was tested using a significance level of 0.05. To compare the means (ie, CML or fAGEs formation using glyoxal and glyoxylic acid), the paired *t* test (two‐tailed, α = 0.05) was used. Any *p* values <0.05 were considered statistically significant for all analyses.

Fitting of kinetic curves was performed using exponential (y = A*exp(−Bx) + C) or linear (y = ax+b) equations.

## Results

### Isolation and preparation of bone matrix proteins for CML quantitation

The quality and the amount of isolated bone matrix proteins determine the outcome and reproducibility of the performed CML‐ELISA. Because bone matrix contains high amount of collagen (up to 90% of organic matrix^(^ ^34^, ^35^ ^)^), this protein is the major component of the isolated protein fraction. Moreover, most of the glycated collagen and bone matrix proteins are isolated in the form of pellet that has to be solubilized. Because the freezing‐and‐thawing procedure (60 minutes at −80°C and 15 minutes at +85°C) is a slow process, several steps of sonication and cooling (90 minutes sonication and 20 minutes on ice) were introduced to speed up the solubilization process. Solubilization of glycated protein pellets was conducted until ~50 to 60 μg of solubilized protein (out of 100 μg initial protein pellet) was obtained for CML ELISA (Table 1).

### Glyoxal facilitates formation of higher levels of CML in bone matrix than glyoxylic acid

**Fig 2:**
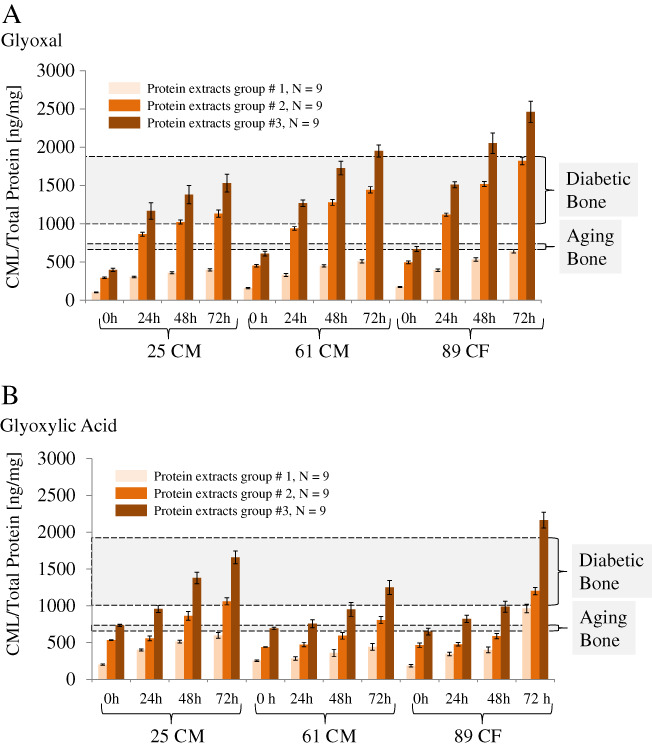
Levels of CML determined in soluble protein fraction (light russet) and solubilized pellet (russet), and their sum (brown) formed using glyoxal (*A*) and glyoxylic acid (*B*). The color bars represent a mean of triplicate and the error bars depict ±SD. Typical levels of CML in diabetic^(^ ^39^, ^40^ ^)^ and aging^(^ ^20^ ^)^ bone are highlighted in gray. Bone matrix proteins were isolated from three bone samples originating from each donor. Protein extracts were divided into three portions for CML‐ELISA giving a total of nine protein extracts for the quantitation of CML. Soluble protein fraction usually contains newly in vivo synthesized collagen and other bone matrix proteins, which are normally glycated at a lower level. In vitro glycation lowers the amount of proteins in the soluble fraction. As high level of glycation decreases protein solubility, the pellet fraction is often used alone to measure extend of glycation in a given tissue, here bone's ECM. Two‐factor ANOVA with replication (α = 0.05) showed that the formation of CML depended on the reaction time (*p* < 0.000) and donor's age (*p* < 0.000,) and the interaction between the two was *p* = 0.011 (glyoxal) and *p* < 0.000 (glyoxylic acid). The Tukey HSD (95% CI) test was used post hoc and showed that the formation of CML using either glyoxal (*p* < 0.000) or glyoxylic acid (*p* < 0.000) was significant after 72 hours. The paired *t* tests (two‐tailed, α = 0.05) showed that there was a significant difference in the CML formation between glyoxal and glyoxylic acid (*p* < 0.000). CF = Caucasian female; CM = Caucasian male.

Experimental results of CML measurements are shown in Fig. 2.^(^ ^20^, ^39^, ^40^ ^)^ Quantitation of CML was performed in the soluble fraction (supernatants) and solubilized collagen pellets (Fig. 2*A*,*B*). The final analyses were done using the total amounts of generated CML, which in each case was the sum of both aforementioned fractions.

Quantitation of the CML levels formed in the extracellular bone matrix revealed that glycation using glyoxal led to the formation of higher levels of CML (Fig. 2*A*) as compared to glyoxylic acid (Fig. 2*B*). All observed differences were statistically significant (*p* < 0.001). For example, the total levels of CML formed within 72 hours, which were calculated as the average of all sums for the three age groups, were greater for glyoxal (1979.7 ± 465.7 ng of CML per mg protein) than for glyoxylic acid (1690.4 ± 459.2 ng of CML per mg protein). Considering the age of donors, the determined levels of CML formed within 72 hours of incubation with glyoxal were 1529.6 ± 131.0 CML/protein [ng/mg]) for the 25‐year‐old CM (25 CM), 1949.9 ± 132.4 CML/protein [ng/mg]) for the 61‐year‐old CM (61 CM), and 2459.6 ± 169.4 CML/protein [ng/mg]) for the 89‐year‐old CF (89 CF), and thus, were typically higher as compared to the corresponding incubation time with glyoxylic acid (1658.6 ± 70.9 ng CML/mg protein for the 25 CM, 1248.0 ± 84.2 ng CML/mg protein for the 61 CM, and 2164.7 ± 91.4 ng CML/mg protein for the 89 CF).

Two‐factor ANOVA with replication (α = 0.05) was employed to make a comparison between the CML formation with glyoxal or glyoxylic acid for reactions conducted for 24, 48, and 72 hours, and donors 25 CM, 61 CM, and 89 CF. This analysis showed that the formation of CML depends on the reaction time (*p* < 0.000) and donor's age (*p* < 0.000,) and the interaction between the two was *p* = 0.011 (glyoxal) and *p* < 0.000 (glyoxylic acid). The Tukey HSD (95% CI) test was used post hoc and showed that the formation of CML using either glyoxal (*p* < 0.000) or glyoxylic acid (*p* < 0.000) was significant after 72 hours. The paired *t* tests (two‐tailed, α = 0.05) were performed for 24, 48, and 72 hours, for donors 25 CM, 61 CM, and 89 CF, as well as for data pooled from the three donors. All the aforementioned *t* test comparisons showed that there was a significant difference in the CML formation between glyoxal and glyoxylic acid (*p* < 0.000).

### Kinetics of the CML formation in bone matrix using glyoxal and glyoxylic acid

**Fig 3:**
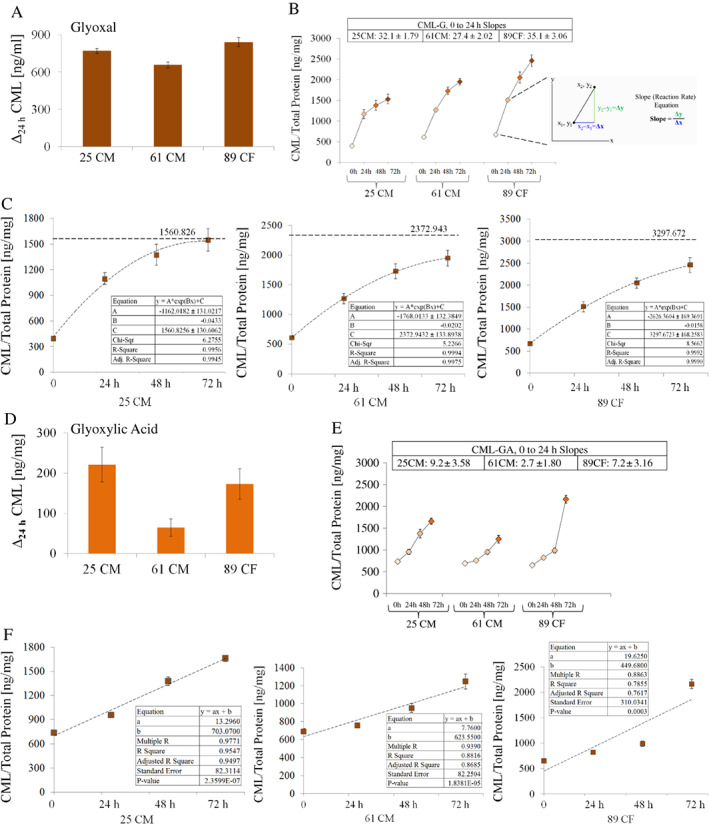
Total CML levels generated in bone matrix using either glyoxal (*A*,*B*) or glyoxylic acid (*D*,*E*). The rate of reaction is the change in the amount of a product (here, CML) per unit time (here, hours). The inset in *B* highlighted in gray shows the slope equation Δy/Δx. The slopes corresponding to the CML formation within the first 24 hours of the reaction time are shown as insets in *B* and *E*. The squares represent a mean of triplicate and the error bars depict ±SD. The exponential curve fittings corresponding to the CML formation using glyoxal are shown in *C*. For all donors, this model predicted continuation of the CML formation well beyond 72 hours. Conversely, the linear curve fittings were the best to describe the formation of CML for all donors when using glyoxylic acid (ie, for 25 CM R^2^ = 95.5%, *p* < 0.000; for 61 CM R^2^ = 88.2%, *p* < 0.000; and for 89 CF R^2^ = 78.6%, *p* < 0.000.) These linear fittings are shown in *F*. Δ_24h_ = a change in the CML formation within the first 24 hours; CF, Caucasian female; CM = Caucasian male.

The key goals of performing kinetic analysis of the CML formation were to evaluate effectiveness of the developed reaction strategy and to capture the difference in the levels of CML formation between glyoxal and glyoxylic acid. This information can be obtained through calculation of the reaction slopes,^(^ ^41^ ^)^ which correspond to the reaction rates (Fig. 3*B*,*E*, Table 2), and which are an established mathematical interpretation of experimental results for chemical/biochemical reactions. Under the developed reaction conditions, the formation of CML occurred primarily between lysines and either glyoxal or glyoxylic acid (ie, carbonyl compounds that preferentially react with lysines^(^ ^22^, ^23^, ^24^, ^25^, ^26^ ^)^).

**Table 2:** Slopes (Reaction Rates) for CML and fAGE Formation Using Glyoxal and Glyoxylic Acid

	Slope (reaction rate)		
Sample	24 hours	48 hours	72 hours
Glyoxal—CML			
25 CM	**32.1 ± 1.79**	8.9 ± 1.59	6.2 ± 0.47
61 CM	**27.4 ± 2.02**	19.2 ± 1.78	9.3 ± 0.37
89 CF	**35.1 ± 3.06**	22.6 ± 2.15	17.0 ± 2.79
Glyoxal—fAGEs			
25 CM	**20.8 ± 4.48**	5.0 ± 5.40	2.2 ± 4.75
61 CM	**31.4 ± 4.70**	6.3 ± 5.76	3.5 ± 7.03
89 CF	**32.2 ± 3.76**	8.1 ± 1.58	3.6 ± 5.77
Glyoxylic acid—CML			
25 CM	**9.2 ± 3.58**	17.5 ± 1.94	11.7 ± 7.33
61 CM	**2.7 ± 1.80**	8.1 ± 2.63	12.4 ± 1.51
89 CF	**7.2 ± 3.16**	6.9 ± 3.39	49.0 ± 1.56
Glyoxylic acid—fAGEs			
25 CM	**17.9 ± 3.45**	2.8 ± 2.94	3.5 ± 5.81
61 CM	**25.7 ± 5.93**	4.6 ± 4.99	4.2 ± 3.86
89 CF	**23.9 ± 5.19**	9.4 ± 4.45	9.5 ± 5.02

The slopes ± SD for 0 to 24 hours calculated according to the equation Δy/Δx are in bold. Initial reaction rate (here, from 0 to 24 hours) is normally used to evaluate the formation of the products of interest. With the increase of reaction time, the reaction rate decreases. Later, such decrease could potentially become skewed because of, for example, depletion of the reactants leading to their imbalance. Considering bone, natural heterogeneity of the tissue could lead to additional differences in the accumulation of glycation products even between samples originating from the matching areas of the same donor.

The graphical representation of the CML reaction kinetics is shown in Fig. 3*B*,*E*; these biomolecular reactions behave like the first‐order reaction. The rate of reaction is the change in the amount of a product (here, CML or fAGEs) per unit time (here, hours) (the inset in Fig. 3*B* highlighted in gray shows the slope equation Δy/Δx). The calculated reaction slopes corresponding to the reaction rates are summarized in Table 2. The respective rates (Table 2) revealed that in the case of glyoxal, the formation of CML was most pronounced within the first 24 hours (Δ_24h_, Fig. 3*A* and the slope inset in the inset panel of Fig. 3*B*) and then, depending on the donor, continued until 72 hours, when it gradually began to decline (eg, caused by a consumption of substrates). In contrast to glyoxal, CML formation using glyoxylic acid was relatively slow within the first 24 hours (Δ_24h_, Fig. 3*D*), and then, markedly increased within the next 48 to 72 hours, reaching the highest level at 72 hours (Fig. 3 *E* and Table 2).

We determined that the exponential curve fitting model (y = A*exp(Bx) + C; y = CML concentration, x = time) was the best to capture the trend of CML formation using glyoxal for all donors (ie, for 25 CM R^2^ = 99.6%; for 61 CM R^2^ = 99.9%; and for 89 CF R^2^ = 99.9%; Fig. 3*C*). The model predicted asymptotic saturation of CML formation at 1560.8 ng of CML per mg protein for donor 25 CM, 2372.9 ng of CML per mg protein for donor 61 CM, and 3297.7 ng of CML per mg protein for donor 89 CF. Conversely, the linear curve fitting model (y = ax+b) was the best to describe the CML formation using glyoxylic acid for all donors (ie, for 25 CM R^2^ = 95.5%, *p* < 0.000; for 61 CM R^2^ = 88.2%, *p* < 0.000; and for 89 CF R^2^ = 78.6%, *p* < 0.000; Fig. 3*F*). The exponential versus linear curve fitting agrees, for example, with chemical differences between glyoxal (eg, contains two functional aldehyde groups) and glyoxylic acid (eg, contains one functional aldehyde group,) and the chemical nature of CML that is formed as a result of simpler lysine modification by carboxymethyl group when compared to other structurally more complex AGEs (eg, pentosidine, vesperlysines, crossline).^(^ ^42^, ^43^, ^44^, ^45^, ^46^, ^47^, ^48^, ^49^, ^50^ ^)^

### Formation of fAGEs is higher with glyoxal than glyoxylic acid

**Fig 4:**
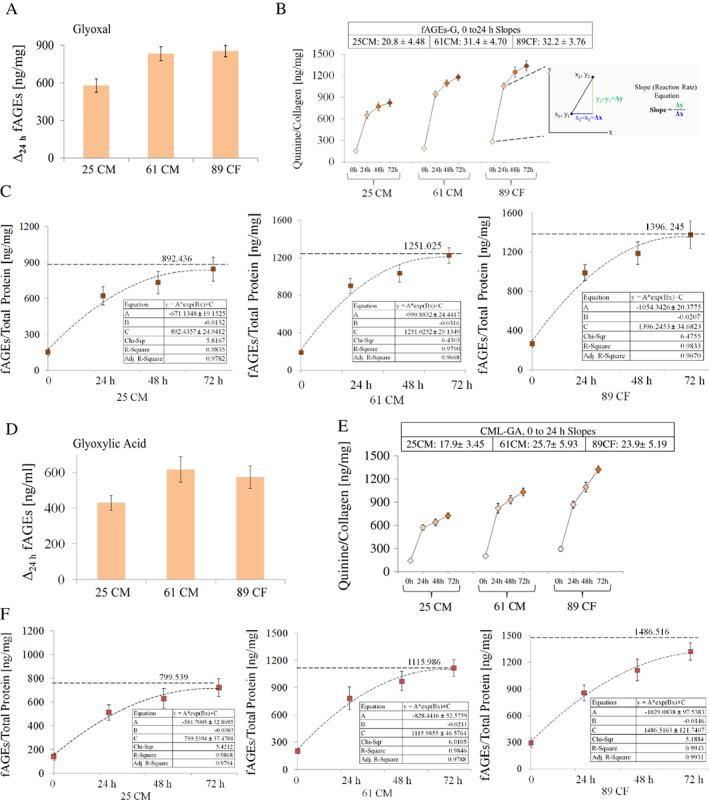
Fluorescent AGE levels generated in bone matrix using either glyoxal (*A*,*B*) or glyoxylic acid (*D*,*E*). The rate of reaction is the change in the amount of a product (here, fAGEs) per unit time (here, hours). The inset in *B* highlighted in gray shows the slope equation Δy/Δx. The slopes corresponding to the fAGE formation within the first 24 hours of the reaction time are inserted in *B* and *E*. The squares represent a mean of triplicate and the error bars depict ±SD. The exponential curve fittings corresponding to the fAGE formation using glyoxal and glyoxylic acid are shown in *C* and *F*. The exponential model predicted early asymptotic saturation of the fAGE formation for all donors. Importantly, the saturation of the fAGE formation coincided with the decline of the corresponding reaction rates (Table 2). Δ_24h_ = a change in the CML formation within the first 24 hours; CF = Caucasian female; CM = Caucasian male.

As in the case of CML formation using glyoxal, the formation of fAGEs occurred at the highest rate within the first 24 hours (Δ_24h_, Fig. 4*A*,*B*; the inset in Fig. 4*B* highlighted in gray shows the slope equation Δy/Δx) but with the lower reaction rates for fAGEs than CML (Table 2). Importantly, the production of fAGEs declined pronouncedly after 24 hours, while the formation of CML continued (Table 2). Initial generation of fAGEs using glyoxylic acid followed the opposite course when compared to glyoxal. At first (ie, within the first 24 hours) fAGEs were formed at a higher rate than CML, and then, the rate of their formation markedly dropped down (Table 2; Δ_24h_, Fig. 4*D*), while the reaction rate for CML continued to be high.

Considering the age of donors, for example, the determined levels of fAGEs that formed within 72 hours with glyoxylic acid were 723.5 ± 74.9 (25 CM), 1031.0 ± 93.2 (61 CM), and 1323.2 ± 97.5 (89 CF) Q/Collagen [ng/mg] (Fig. 4*E*), and thus, were relatively similar to the levels generated when using glyoxal, ie, 823.4 ± 99.8 (25 CM), 1179.1 ± 80.5 (61 CM), and 1336.3 ± 138.7 (89 CF) Q/Collagen [ng/mg] (Fig. 4*B*).

Two‐factor ANOVA with replication (α = 0.05) was employed to make comparisons between the fAGEs formation with glyoxal or glyoxylic acid for reactions conducted for 24, 48, and 72 hours, and donors 25 CM, 61 CM, and 89 CF. This analysis showed that the formation of fAGEs depends on the reaction time (*p* < 0.000) and donor's age (*p* < 0.000), and the interaction between the two was *p* = 0.906 (glyoxal) and *p* = 0.129 (glyoxylic acid). The Tukey HSD (95% CI) test was used as post hoc and showed that the formation of fAGEs using glyoxal (*p* = 0.280) was not significant, but for glyoxylic acid (*p* = 0.013) was still significant after 72 hours. The paired *t* tests (two‐tailed, α = 0.05) were performed for 24, 48, and 72 hours, and donors 25 CM, 61 CM, and 89 CF, as well as for data pooled from the three donors. All the aforementioned *t* test comparisons showed that there was a significant difference in the fAGEs formation between glyoxal and glyoxylic acid (*p* < 0.000).

We also determined that for all donors, the exponential curve fitting model (y = A*exp(Bx) + C; y = fAGE concentration, x = time) was the best to describe the formation of fAGEs using glyoxal (ie, for 25 CM R^2^ = 98.3%; for 61 CM R^2^ = 97.9%; and for 89 CF R^2^ = 98.3%; Fig. 4*C*) and glyoxylic acid (ie, for 25 CM R^2^ = 98.7%; for 61 CM R^2^ = 98.5%; and for 89 CF R^2^ = 99.4%; Fig. 4*F*). The models predicted relatively similar asymptotic saturation of fAGE formation for glyoxylic acid (ie, at 799.5 fAGEs/Total Protein [ng/mg] for donor 25 CM, 1116.0 fAGEs/Total Protein [ng/mg] for donor 61 CM, and 1486.5 fAGEs/Total Protein [ng/mg] for donor 89 CF, and glyoxal (ie, at 892.4 fAGEs/Total Protein [ng/mg] for 25 CM, 1251.0 fAGEs/Total Protein [ng/mg] for 61 CM, and 1396.2 fAGEs/Total Protein [ng/mg] for 89 CF). Notably, the saturation of fAGE formation coincided with the decline of the corresponding reaction rates (Table 2). Interestingly, formation of fAGEs with either glyoxal or glyoxylic acid followed the exponential curve fitting model. Thus, there is a difference between the models describing formation of CML (ie, the linear model) and fAGEs (ie, the exponential model) when using glyoxylic acid as the glycation substrate. Such difference could be explained by, for example, the formation of many structurally more complex AGEs than CML alone.

## Discussion

In the late 1980s and early 1990s, it was established that CML levels increase with age, for example, in human lenses,^(^ ^1^ ^)^ human skin,^(^ ^47^ ^)^ and in diabetes, where the severity of diabetic complications correlated with the CML levels.^(^ ^48^ ^)^ Recently, it was shown that ECM of human bone contains CML.^(^ ^20^ ^)^ Both CML and pentosidine are the early glycoxidation products.^(^ ^45^, ^46^ ^)^ Comparison of CML levels with the levels of another important AGE known to be present in bone, pentosidine (PEN),^(^ ^8^, ^37^, ^51^ ^)^ showed significantly more CML than PEN in human bone tissue.^(^ ^20^ ^)^ The authors proposed that the increased abundance of CML relative to PEN could provide an alternative, and potentially more sensitive method, for assessing AGE accumulation in bone tissue. Still, the relative levels of formed CML over other AGEs in bone, their ratios and functional relevance to bone health as well as other tissue types remain to be elucidated.

To better understand the role of the Maillard reaction products in bone‐related disorders and resulting fragility fractures, and to develop effective therapeutic approaches to prevent formation or to remove AGEs from bone, it is essential to understand the effects of specific AGEs on the quality of bone organic matrix. Thus, the goal of this study was to develop a strategy promoting preferential formation of CML in the ECM of bone. We aimed to generate the CML levels of clinical relevance, for example, the levels which would correspond to those observed in vivo in such conditions as aging,^(^ ^17^ ^)^ diabetes,^(^ ^2^, ^18^ ^)^ and renal failure.^(^ ^19^ ^)^

We tested several reaction parameters (eg, reactants, reactant concentrations, temperature, and/or reaction time) and focused on the most promising ones such as: (i) small molecule precursors of CML, which are formed in vivo under oxidative and carbonyl stress conditions, and (ii) the length of incubation time, both of which should effectively control the levels of CML produced in bone matrix. After initial assessment, we selected two compounds that are normally present in the human body^(^ ^2^, ^48^, ^52^, ^53^, ^54^ ^)^—glyoxal and glyoxylic acid—to enhance CML formation over other AGEs under controlled reaction conditions in order to convert healthy bone characterized by lower levels of CML and bulk fAGEs into the one mimicking the aforementioned bone‐related disorders (ie, aging, diabetes, and renal failure). We believe that the developed strategy opens new avenues for studies on mechanistic aspects of bone matrix glycation, including bone cell responses, mineralization, and biomechanical properties of bone, which otherwise would not be possible to conduct in vivo. Thus for the first time, we report here a new strategy for enhanced in vitro formation of CML in bone.

Our results indicate that glyoxal is a potent glycation agent because of its capacity to glycate mineralized bone faster and to a higher degree, especially when compared to glyoxylic acid, which alone is an effective glycation reagent. Such significant formation of CML during bone matrix glycation can be explained, for example, by facile reaction of highly reactive glyoxal (contains two aldehyde groups, Fig. 1*A*) with free functional groups (−NH_2_) of lysine residues.

We also observed an increase of the fAGEs formation, which was expected, because generation of other AGOEs through spontaneous non‐enzymatic process (both in vivo and in vitro when using biological materials) cannot be completely uncoupled from the CML formation. The reason is the presence of other amino acids with free functional groups in bone matrix (eg, arginine, tyrosine, cysteine), which are known to react with glyoxal, glyoxylic acid, and other reactive carbonyls, but not to the same degree as with lysine residues.^(^ ^22^, ^26^ ^)^ Such accompanying formation of fAGEs could be considered as a limitation of the developed strategy. However, our strategy permits adequate separation of the CML formation from other fAGEs through the control of reaction time. Our kinetic studies of the initial phase of the CML versus fAGEs formation and analysis of the corresponding reaction rates revealed that under our reaction conditions, the formation of CML and fAGEs followed different courses for both glyoxal and glyoxylic acid. In general, the two compounds favored formation of CML over fAGEs not only in regard to the generated CML amounts, but also in regard to the reaction time span, which was clearly different for CML and fAGEs. Our data show that saturation of fAGE formation occurred fast (ie, within the first 24 hours) and then markedly slowed down, while the formation of CML continued at high rate. Therefore, it can be safely assumed that beyond the experimental reaction time of 48 hours or more, the formation of fAGEs becomes minor. Moreover, we also established that the exponential curve fitting models were the best to capture the trends of the CML (Fig. 3*C*) and fAGE (Fig. 4*C*) formation using glyoxal for all donors. In the case of glyoxylic acid, the CML formation was best described by the linear model (Fig. 3*F*) and fAGEs by exponential model (Fig. 4*F*) for all donors. The models predicted asymptotic saturation of fAGE formation for each donor, but not for CML because the formation of CML continued. In summary, quantitative formation of CML and fAGEs can be separated experimentally by simple diversification of the reaction times.

We propose that our newly developed strategy could be used, for example, to elucidate the role of CML in biomechanical properties of bone tissue or cell responses to bone matrix altered by reactive carbonyls. Moreover, in combination with ribosylation or glucosylation,^(^ ^8^, ^16^ ^)^ the two methods that are commonly used for bone matrix glycation and are known to generate AGEs,^(^ ^8^, ^16^ ^)^ one could further expand the studies on the mechanistic aspects of glycation in different diseases. For example, it is likely that early and late stages of the diabetic condition may differ in the levels of formed specific AGEs (eg, CML, PEN) as well as in the ratios between such specific AGEs and other AGEs measured “in bulk.” We posit that confirming the aforementioned hypothesis will allow the use of certain AGE combinations as a diagnostic tool for bone fragility while providing new information on etiology and potential therapeutic intervention, for example, for diabetic and age‐related fractures.

As hypothesized, the quality of bone matrix and mineral—known to change with age^(^ ^8^, ^32^, ^33^ ^)^—influenced the development of our in vitro reaction conditions and the overall glycation process. An approximately 30‐year age gap between the selected donors was included in the experimental design to capture the age‐related differences in the quality of bone matrix and mineral^(^ ^8^, ^32^, ^33^ ^)^ that may influence the outcomes of the performed in vitro glycation. An interesting observation was that glycation using glyoxal or glyoxylic acid produced higher levels of CML for older donors than the young donor. The reason for generating higher CML levels in cortical bone samples from older donors could be attributed to, for example, the age‐related structural differences between the samples. It was shown previously that the amount and quality of mineral phase decreases with aging and this significantly increases the porosity of bone tissue.^(^ ^55^ ^)^ Increased porosity exposes more amino acids on protein surfaces to the local microenvironment, and thus, leads to more efficient matrix glycation both in vivo and in vitro.^(^ ^8^ ^)^

**Fig 5:**
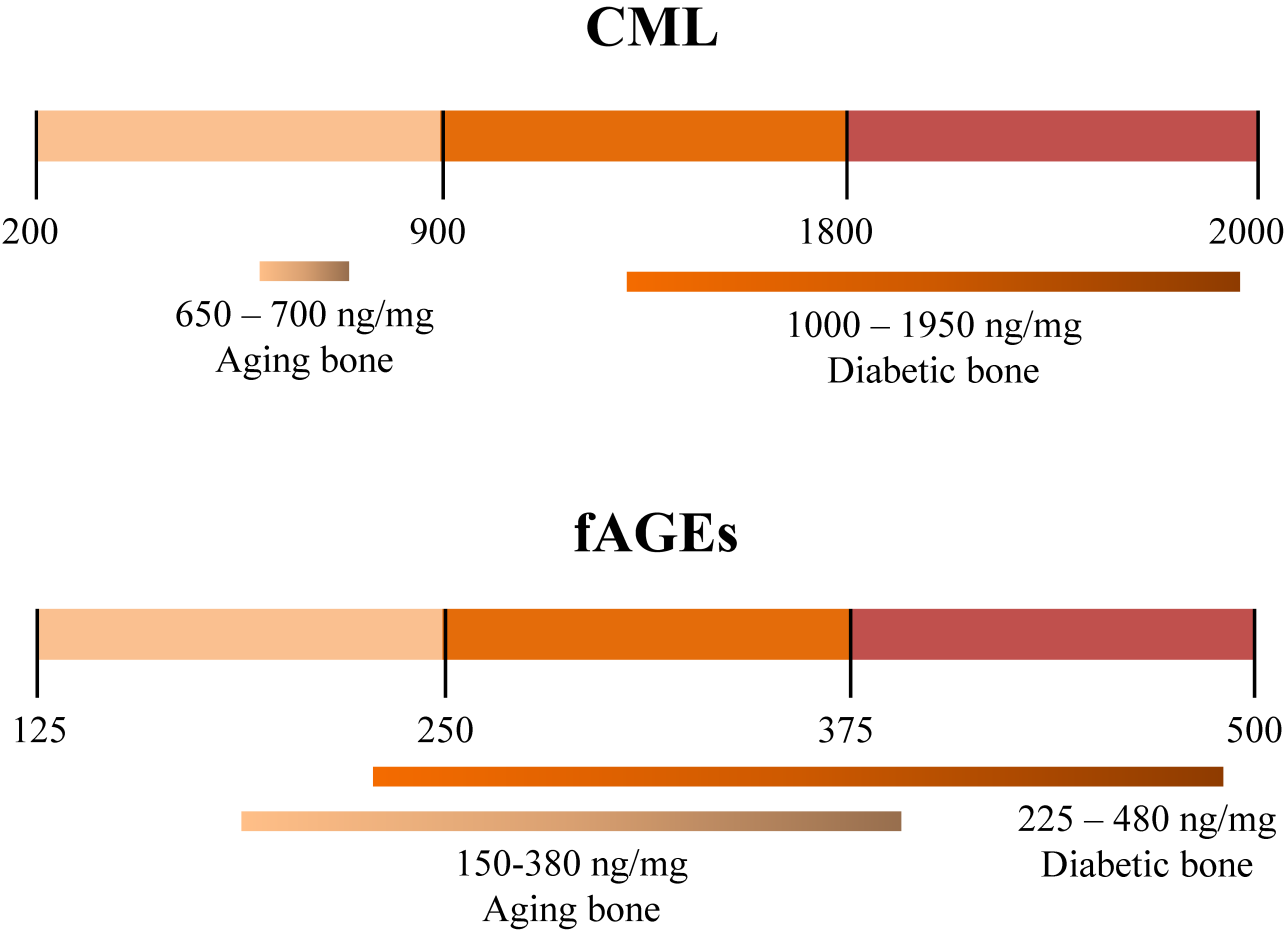
Examples of the CML levels that can be generated using the developed strategy. The use of either glyoxal or glyoxylic acid helps to diversify generation of CML levels in tissues of interest. The levels of CML shown for aging bone are from this study. The levels of CML observed in different human tissues^(^ ^2^, ^44^ ^)^ including bone^(^ ^20^, ^39^, ^40^ ^)^ are from the published literature. The levels of fAGEs are shown for comparison purposes.

Our studies also revealed a surprisingly fast conversion of lysine residues into CML when using glyoxal and the conditions that favor the Maillard reaction. Importantly, such favorable conditions (ie, increased cellular and systemic oxidative stress) for CML formation are observed, for example, in osteoporosis and diabetes.^(^ ^52^, ^53^, ^54^ ^)^ We showed that after 72 hours, which is a very short glycation time when compared to a standard glycation using ribose (~7 to 38 days^(^ ^16^ ^)^), the generated levels of CML in bone matrix corresponded to those observed in aging^(^ ^2^, ^18^ ^)^ bone (eg, ~650 to 700 CML/total protein [ng/mg]) and different tissues of T2DM patients (eg, ~1000 to 1950 CML/total protein [ng/mg])^(^ ^2^, ^18^, ^39^, ^40^ ^)^ (Figs. 2 and 5). These data highlight the capability of our technique to generate both CML^(^ ^2^, ^18^, ^39^, ^40^ ^)^ and AGE^(^ ^56^, ^57^ ^)^ levels seen in vivo with diabetes and aging. More importantly, our work also shows why CML levels increase dramatically with diabetes as compared to aging bone. Thus, a technique to elevate or generate specific CML levels in vitro should help to understand its adventitious effects on bone.

Most of the studies on the role and mechanistic aspects of specific AGEs in biomechanical properties of bone at different hierarchical levels of its structure are performed in vitro. Those conducted at the microscale and nanoscale levels typically focus on the biomechanical function of bone matrix proteins such as collagen and non‐collagenous matrix proteins (NCPs).^(^ ^58^, ^59^, ^60^ ^)^ The mechanistic implications of the CML presence in bone matrix are yet to be unraveled.

**Fig 6:**
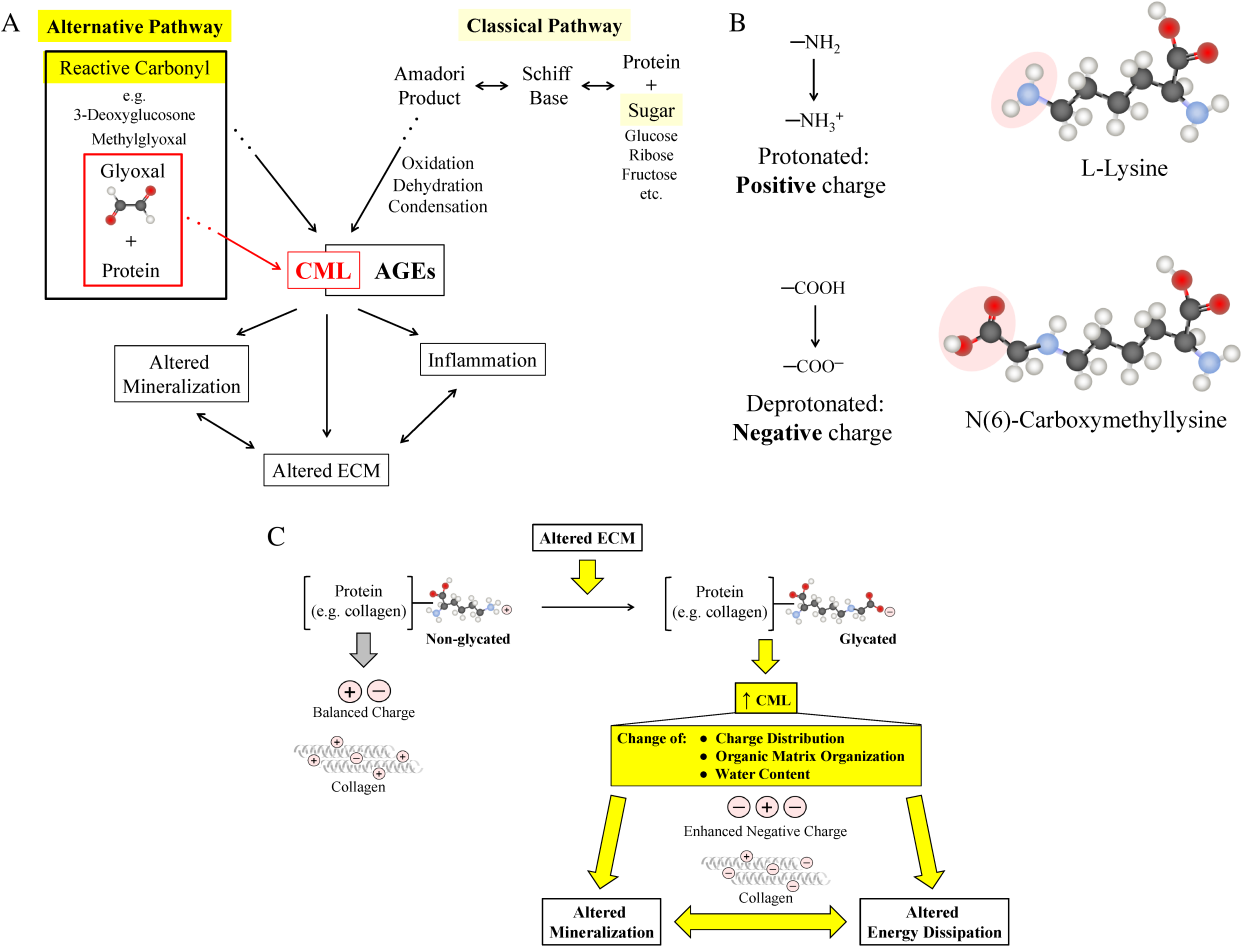
Proposed principles (*A*,*B*) and consequences (*C*) of CML formation in bone. (*A*) Enhancement of CML formation was achieved using reactive carbonyls (eg, glyoxal). (*B*) Formation of CML leads to a change of charge for a given protein from positive (attributed to L‐lysine residues) to negative (attributed to CML). (*C*) Consequences of the increased CML content in bone's ECM: (i) CML alters native charge distribution and molecular organization of extracellular matrix; (ii) more negative charge impacts such processes and matrix mineralization (eg, attracts more calcium ions, initiates and facilitates formation of hydroxyapatite crystals) and matrix function (eg, energy dissipation).

We reason that in addition to inducing inflammation and related with it consequences, CML also modifies ECM properties and these changes lead to altered mineralization (Fig. 6*A*). Our hypothesis that CML could impact several characteristics of organic and mineral components of bone tissue is based on the fact that the positive charge of L‐lysine is replaced by the negative charge of CML (Fig. 6*B*). Thus, modification of ECM proteins' charge distribution would impact molecular organization of the organic matrix, in particular, the most abundant protein in bone's ECM, collagen (Fig. 6*C*). Charge, for example, is known to be critical to the process of healthy mineralization^(^ ^38^, ^61^, ^62^ ^)^ and energy dissipation,^(^ ^58^, ^59^, ^60^, ^63^ ^)^ both of which are known to affect bone fragility.

Formation of CML leads to a change of charge for a given protein from positive (attributed to L‐lysine residues) to negative (attributed to CML). As a result, this will lead to alterations of native charge distribution and molecular organization of ECM. More negative charge will impact such process as matrix mineralization (eg, attracts more calcium ions, initiates and facilitates formation of altered hydroxyapatite crystals) and matrix function (eg, energy dissipation). Thus, our new in vitro glycation strategy will facilitate investigation of these mechanistic aspects, following the generation of the desired/diverse levels of CML in bone matrix.

In conclusion, we developed an in vitro strategy facilitating controlled formation of the desired/higher levels of CML over other AGEs in human cortical bone using either glyoxal or glyoxylic acid. Separation of the effects of CML imposed by this AGE on bone tissue from those caused by other AGEs could be achieved experimentally by a simple diversification of the in vitro reaction times. The developed strategy could serve as an experimental tool to study the mechanistic aspects of CML in bone matrix, and in general, could open new avenues for studies on the role of a variety of specific AGEs; eg, in different oxidative stress‐related diseases. Because the used molecules are not only generated in the human body but also are present in the environment (eg, drinking water, hygiene products and cosmetics, tobacco smoke, residential log fire, and vehicle exhaust), our studies may help to understand environmentally‐related development of certain bone and other organs pathologies that are related to oxidative stress and biological in vivo glycation.

## Conflicts of Interest

The authors state that they have no conflicts of interest.

### Peer Review

The peer review history for this article is available at https://publons.com/publon/10.1002/jbm4.10548.

## References

[1] Dunn JA , Patrick JS , Thorpe SR , Baynes JW . Oxidation of glycated proteins: age‐dependent accumulation of N'‐(carboxymethyl)lysine in lens proteins. Biochemistry. 1989;28(24):9464‐9468. 2514802 10.1021/bi00450a033

[2] Dyer DG , Dunn JA , Thorpe SR , et al. Accumulation of Maillard reaction products in skin collagen in diabetes and aging. J Clin Invest. 1993;91(6):2463‐2469. 8514858 10.1172/JCI116481 PMC443306

[3] Sell DR , Monnier VM . Structure elucidation of a senescence cross‐link from human extracellular matrix. Implication of pentoses in the aging process. J Biol Chem. 1989;264:21597‐21602. 2513322

[4] Lederer MO , Bühler HP . Cross‐linking of proteins by Maillard processes—characterization and detection of a lysine‐arginine cross‐link derived from D‐glucose. Bioorg Med Chem. 1999;7:1081‐1088. 10428377 10.1016/s0968-0896(99)00040-1

[5] Nakamura K , Nakazawa Y , Ienaga K . Acid‐stable fluorescent advanced glycation end products: vesperlysines A, B, and C are formed as crosslinked products in the Maillard reaction between lysine or proteins with glucose. Biochem Biophys Res Commun. 1997;232:227‐230. 9125137 10.1006/bbrc.1997.6262

[6] Vistoli G , De Maddis D , Cipak A , Zarkovic N , Carini M , Aldini G . Advanced glycoxidation and lipoxidation end products (AGEs and ALEs): an overview of their mechanisms of formation. Free Radic Res. 2013;47(Sup1):3‐27. 10.3109/10715762.2013.815348. 23767955

[7] Chaudhuri J , Bains Y , Guha S , et al. The role of advanced glycation end products in aging and metabolic diseases: bridging association and causality. Cell Metab. 2018;28(3):337‐352. 10.1016/j.cmet.2018.08.014. 30184484 PMC6355252

[8] Sroga GE , Siddula A , Vashishth D . Glycation of human cortical and cancellous bone captures differences in formation of Maillard reaction products between glucose and ribose. PLoS One. 2015;10(2):e0117240. 10.1371/journal.pone.0117240. 25679213 PMC4334514

[9] Grandhee SK , Monnier VM . Mechanism of formation of the Maillard protein cross‐link pentosidine. Glucose, fructose, and ascorbate as pentosidine precursors. J Biol Chem. 1991;266(18):11649‐11653. 1904866

[10] Igaki N , Sakai M , Hata H , Oimomi M , Baba S , Kato H . Effects of 3‐deoxyglucosone on the Maillard reaction. Clin Chem. 1990;36:631‐634. 2157564

[11] Boesten DMPHJ , Elie AGIM , Drittij‐Reijnders MJ , den Hartog GJM , Bast A . Effect of N‐carboxymethyllysine on oxidative stress and the glutathione system in beta cells. Toxicol Rep. 2014;1:973‐980. 10.1016/j.toxrep.2014.06.003. 28962310 PMC5598217

[12] Coughlan MT , Yap FYT , Tong DCK , et al. Advanced glycation end products are direct modulators of β‐cell function. Diabetes. 2011;60(10):2523‐2532. 10.2337/db10-1033. 21911745 PMC3178291

[13] McCarthy AD , Etcheverry SB , Bruzzone L , Cortizo AM . Effects of advanced glycation end‐products on the proliferation and differentiation of osteoblast‐like cells. Mol Cell Biochem. 1997;170:43‐51. 9144317 10.1023/a:1006816223292

[14] Meng HZ , Zhang WL , Liu F , Yang MW . Advanced glycation end products affect osteoblast proliferation and function by modulating autophagy via the receptor of advanced glycation end products/Raf protein/mitogen‐activated protein kinase/extracellular signal‐regulated kinase kinase/extracellular signal‐regulated kinase (RAGE/Raf/MEK/ERK) pathway. J Biol Chem. 2015;290(47):28189‐28199. 10.1074/jbc.M115.669499. 26472922 PMC4653677

[15] Li Z , Li C , Zhou Y , et al. Advanced glycation end products biphasically modulate bone resorption in osteoclast‐like cells. Am J Physiol Endocrinol Metab. 2016;310:E355‐E366. 10.1152/ajpendo.00309.2015. 26670486

[16] Vashishth D , Gibson GJ , Khoury JI , Schaffler MB , Kimura J , Fyhrie DP . Influence of nonenzymatic glycation on biomechanical properties of cortical bone. Bone. 2001;28:195‐201. 11182378 10.1016/s8756-3282(00)00434-8

[17] Semba RD , Fink JC , Sun K , Bandinelli S , Guralnik JM , Ferrucci L . Carboxymethyl‐lysine, an advanced glycation end product, and decline of renal function in older community‐dwelling adults. Eur J Nutr. 2009;48:38‐44. 10.1007/s00394-008-0757-0. 19031098 PMC2637810

[18] Franke S , Dawczynski J , Strobel J , Niwa T , Stahl P , Stein G . Increased levels of advanced glycation end products in human cataractous lenses. J Cataract Refract Surg. 2003;29(5):998‐1004. 12781289 10.1016/s0886-3350(02)01841-2

[19] Wagner Z , Wittmann I , Mazák I , et al. N(epsilon)‐(carboxymethyl)lysine levels in patients with type 2 diabetes: role of renal function. Am J Kidney Dis. 2001;38(4):785‐791. 10.1053/ajkd.2001.27695. 11576882

[20] Thomas CJ , Cleland TP , Sroga GE , Vashishth D . Accumulation of carboxymethyl‐lysine (CML) in human cortical bone. Bone. 2018;110:128‐133. 10.1016/j.bone.2018.01.028. 29408699 PMC5878737

[21] Barzilay JI , Bůžková P , Zieman SJ , et al. Circulating levels of carboxy‐methyl‐lysine (CML) are associated with hip fracture risk: the Cardiovascular Health Study. J Bone Miner Res. 2014;29(5):1061‐1066. 24877243 10.1002/jbmr.2123 PMC4523135

[22] Glomb MA , Monnier VM . Mechanism of protein modification by glyoxal and glycolaldehyde, reactive intermediates of the Maillard reaction. J Biol Chem. 1995;270(17):10017‐10026. 7730303 10.1074/jbc.270.17.10017

[23] Fu MX , Requena JR , Jenkins AJ , Lyons TJ , Baynes JW , Thorpe SR . The advanced glycation end product N‐ε‐(carboxymethyl)lysine, is a product of both lipid peroxidation and glycoxidation reactions. J Biol Chem. 1996;271(17):9982‐9986. 8626637 10.1074/jbc.271.17.9982

[24] Ahmed MU , Brinkmann‐Frye E , Degenhardt TP , Thorpe SR , Baynes JW . N‐ε‐(carboxyethyl)lysine, a product of chemical modification of protein by methylglyoxal, increases with age in human lens proteins. Biochem J. 1997;324:565‐570. 9182719 10.1042/bj3240565 PMC1218467

[25] Baynes JW , Thorpe SR . Glycoxidation and lipoxidation in atherogenesis. Free Radic Biol Med. 2000;28(12):1708‐1716. 10946212 10.1016/s0891-5849(00)00228-8

[26] Singh R , Barden A , Mori T , Beilin L . Advanced glycation end products: a review. Diabetologia. 2001;44:129‐146. 11270668 10.1007/s001250051591

[27] Namiki M , Hayashi T . A new mechanism of the Maillard reaction involving sugar fragmentation and free radical formation. In Waller GR , Feather MS , eds. The Maillard Reaction in Foods and Nutrition. ACS Symposium Series 215. American Chemical Society; 1983 pp 21‐46.

[28] Namiki M . Chemistry of Maillard reactions: recent studies on the browning reaction mechanism and the development of antioxidants and mutagens. Adv Food Res. 1988;32:115‐184. 3075879 10.1016/s0065-2628(08)60287-6

[29] Semchyshyn HM . Fructation in vivo: detrimental and protective effects of fructose. Biomed Res Int. 2013;2013:343914. 10.1155/2013/343914. 23984346 PMC3741926

[30] Booth ED , Dofferhoff O , Boogaard PJ , Watson WP . Comparison of the metabolism of ethylene glycol and glycolic acid in vitro by precision‐cut tissue slices from female rat, rabbit and human liver. Xenobiotica. 2004;34(1):31‐48. 10.1080/00498250310001624636. 14742135

[31] Scientific Committee on Consumer Products (SCCP) . European Commission Health & Consumer Protection Directorate‐General, Directorate C ‐ Public Health and Risk Assessment C7 ‐ Risk assessment SCCP/0881/05 (2005) Opinion on Glyoxal. 4th Plenary, June 21, 2005:54. Available from: https://ec.europa.eu/health/ph_risk/committees/04_sccp/docs/sccp_o_023.pdf.

[32] Sroga GE , Vashishth D . Effects of bone matrix proteins on fracture and fragility in osteoporosis. Curr Osteoporos Rep. 2012;10(2):141‐150. 10.1007/s11914-012-0103-6. 22535528 PMC3375270

[33] Aerssens J , Dequeker J , Mbuyi‐Muamba JM . Bone tissue composition: biochemical anatomy of bone. Clin Rheumatol. 1994;13(Suppl 1):54‐62. 7750243

[34] Boskey AL . Bone composition: relationship to bone fragility and antiosteoporotic drug effects. Bonekey Rep. 2013;2:447. 10.1038/bonekey.2013.181. 24501681 PMC3909232

[35] Feng X . Chemical and biochemical basis of cell‐bone matrix interaction in health and disease. Curr Chem Biol. 2009;3(2):189‐196. 10.2174/187231309788166398. 20161446 PMC2790195

[36] Sroga GE , Karim L , Colón W , Vashishth D . Biochemical characterization of major bone‐matrix proteins using nanoscale‐size bone samples and proteomics methodology. Mol Cell Proteomics. 2011;10(9):1‐12. 10.1074/mcp.M110.006718. PMC3186195 21606484

[37] Sroga GE , Vashishth D . UPLC methodology for identification and quantitation of naturally fluorescent crosslinks in proteins: a study of bone collagen. J Chromatogr B Analyt Technol Biomed Life Sci. 2011;879:379‐385. 10.1016/j.jchromb.2010.12.024. PMC3037828 21242109

[38] Sroga GE , Vashishth D . Phosphorylation of extracellular bone matrix proteins declines with age and contributes to bone fragility. J Bone Mineral Res. 2018;33(12):2214‐2229. 10.1002/jbmr.3552. 30001467

[39] Wölfel EM , Jähn K , Milovanovic P , et al. Individuals afflicted with type 2 diabetes mellitus show lower femoral endocortical Sclerostin expression along with higher fluorescent advanced glycation endproducts. J Bone Miner Res. 2019;34(Suppl 1):284‐285.

[40] Wölfel EM , Jähn K , Schmidt FN , et al. Individuals with type 2 diabetes mellitus show a dimorphic pattern of femoral bone quality affecting the risk of fracture. Bone. 2020;140:115556. 10.1016/j.bone.2020.115556. 32730921 PMC12831507

[41] Lower S . Chemical kinetics and dynamics. Chapter 17.2: Reaction rates typically change with time. Chem1 Virtual Textbook; LibreTexts.^TM^ 2021. Available from: https://chem.libretexts.org/@go/page/46069.

[42] Perrone A , Giovino A , Benny J , Martinelli F . Advanced glycation end products (AGEs): biochemistry, signaling, analytical methods, and epigenetic effects. Oxid Med Cell Longev. 2020;2020:3818196. 10.1155/2020/3818196. 32256950 PMC7104326

[43] Brinkmann‐Frye E , Degenhardt TP , Thorpe SR , Baynes JW . Role of the Maillard reaction in aging of tissue proteins. Advanced glycation end product‐dependent increase in imidazolinum cross‐links in human lens proteins. J Biol Chem. 1998;273(30):18714‐18719. 10.1074/jbc.273.30.18714. 9668043

[44] Avery NC , Bailey AJ . Enzymic and non‐enzymic cross‐linking mechanisms in relation to turnover of collagen: relevance to aging and exercise. Scand J Med Sci Sports. 2005;15(4):231‐240. 10.1111/j.1600-0838.2005.00464.x. 15998340

[45] Odetti P , Rossi S , Monacelli F , et al. Advanced glycation end products and bone loss during aging. Ann N Y Acad Sci. 2005;1043:710‐717. 10.1196/annals.1333.082. 16037297

[46] Saito M , Marumo K . Effects of collagen crosslinking on bone material properties in health and disease. Calcif Tissue Int. 2015;97(3):242‐261. 10.1007/s00223-015-9985-5. 25791570

[47] Dunn JA , McCance DR , Thorpe SR , Lyons TJ , Baynes JW . Age‐dependent accumulation of N epsilon‐(carboxymethyl)lysine and N epsilon‐(carboxymethyl)hydroxylysine in human skin collagen. Biochemistry. 1991;30(5):1205‐1210. 1899338 10.1021/bi00219a007

[48] McCance DR , Dyer DG , Dunn JA , et al. Maillard reaction products and their relation to complications in insulin‐dependent diabetes mellitus. J Clin Invest. 1993;91:2470‐2478. 8514859 10.1172/JCI116482 PMC443307

[49] Chellan P , Nagaraj RH . Early glycation products produce pentosidine cross‐links on native proteins: novel mechanism of pentosidine formation and propagation of glycation. J Biol Chem. 2001;276(6):3895‐3903. 10.1074/jbc.M008626200. 11076948

[50] Paradela‐Dobarro B , Rodiño‐Janeiro BK , Jana Alonso J , et al. Key structural and functional differences between early and advanced glycation products. J Mol Endocrinol. 2016;56(1):23‐37. 10.1530/JME-15-0031. 26581238

[51] Viguet‐Carrin S , Roux JP , Arlot ME , et al. Contribution of the advanced glycation end product pentosidine and of maturation of type I collagen to compressive biomechanical properties of human lumbar vertebrae. Bone. 2006;39(5):1073‐1079. 10.1016/j.bone.2006.05.013. 16829221

[52] Rehman K , Akash MSH . Mechanism of generation of oxidative stress and pathophysiology of type 2 diabetes mellitus: how are they interlinked? J Cell Biochem. 2017;118:3577‐3585. 10.1002/jcb.26097. 28460155

[53] Brings S , Fleming T , Freichel M , Muckenthaler MU , Herzig S , Nawroth PP . Dicarbonyls and advanced glycation end‐products in the development of diabetic complications and targets for intervention. Int J Mol Sci. 2017;18:984. 10.3390/ijms18050984. PMC5454897 28475116

[54] Luc KA , Schramm‐Luc A , Guzik TJ , Mikolajczyk TP . Oxidative stress and inflammatory markers in prediabetes and diabetes. J Physiol Pharmacol. 2019;70(6):809‐824. 10.26402/jpp.2019.6.01. 32084643

[55] Jilka RL , O'Brien CA , Roberson PK , Bonewald LF , Weinstein RS , Manolagas SC . Dysapoptosis of osteoblasts and osteocytes increases cancellous bone formation but exaggerates cortical porosity with age. J Bone Miner Res. 2014;29:103‐117. 23761243 10.1002/jbmr.2007 PMC3823639

[56] Gundberg CM , Anderson M , Dickson I , Gallop PM . “Glycated” osteocalcin in human and bovine bone. The effect of age. J Biol Chem. 1986;261(31):14557‐14561. 3490475

[57] Sroga GE , Vashishth D . Glycated osteocalcin. J Bone Miner Res. 2013;28(Suppl 1) [Poster session presented at: Annual Meeting American Society for Bone and Mineral Research (ASBMR); 2013 Oct 4–7; Baltimore, MD, USA; Presentation Number: MO0102]. p. S365. Available from: https://www.asbmr.org/education/AbstractDetail?aid=afbf0f80‐40a2‐49cb‐a7d5‐8626b2d53b02.

[58] Fantner GE , Hassenkam T , Kindt JH , et al. Sacrificial bonds and hidden length dissipate energy as mineralized fibrils separate during bone fracture. Nat Mater. 2005;4:612‐616. 10.1038/nmat1428. 16025123

[59] Fantner GE , Adams J , Turner P , et al. Nanoscale ion mediated networks in bone: osteopontin can repeatedly dissipate large amounts of energy. Nano Lett. 2007;7:2491‐2498. 17645366 10.1021/nl0712769

[60] Tavacol M , Vughan TJ . The structural role of osteocalcin in bone biomechanics and its alteration in Type‐2 diabetes. Sci Rep. 2020;10:17321. 10.1038/s41598-020-73141-w. 33057142 PMC7560881

[61] Boskey AL . Bone mineralization. In Cowin SC , ed. Bone Biomechanics, vol. 3. CRC Press; 2001 pp 5.1‐5.34.

[62] Boivin G , Meunier PJ . The degree of mineralization of bone tissue measured by computerized quantitative contact microradiography. Calcif Tissue Int. 2002;70:503‐511. 12019458 10.1007/s00223-001-2048-0

[63] Poundarik AA , Diab T , Sroga GE , et al. Dilatational band formation in bone. Proc Natl Acad Sci U S A. 2012;109(47):19178‐19183. 10.1073/pnas.1201513109. 23129653 PMC3511118

